# Supplementary material for: Temporal control of human DNA replication licensing by CDK4/6-RB signalling and chemical genetics
Source: Nat Commun. 2025 Sep 12;16:8268. doi: 10.1038/s41467-025-63669-8 (PMC12432183; doi:10.1038/s41467-025-63669-8)
Supplement: Supplementary file 1 — Supplementary Information [file 41467_2025_63669_MOESM1_ESM.pdf]

Supplementary information

Temporal control of human DNA replication licensing by CDK4/6-RB signalling and chemical genetics

Anastasia Sosenko Piscitello[1,#], Ann-Sofie Nilsson[2,#], Michael Hawgood[2,#], Abid H. Sayyid[2,3], Vasilis S. Dionellis[1], Giovanni Giglio[2], Bruno Urién[2], Pratikiran Bajgain[2], Sotirios G. Ntallis[1], Jiri Bartek[2,4*], Thanos D. Halazonetis[1*] & Bennie Lemmens[2*]

[1] Department of Molecular and Cellular Biology, University of Geneva, Geneva, Switzerland

[2] Department of Medical Biochemistry and Biophysics, Karolinska Institutet, Science for Life Laboratory, Stockholm, Sweden

[3] Department of Civil, Environmental and Natural Resources Engineering, Luleå University of Technology

[4] Danish Cancer Institute, Copenhagen, Denmark

[#] These authors contributed equally to this study.

*Correspondence to: bennie.lemmens@ki.se, thanos.halazonetis@unige.ch, jb@cancer.dk

This file contains Supplementary Figures 1-12 and Supplementary table 1, 2

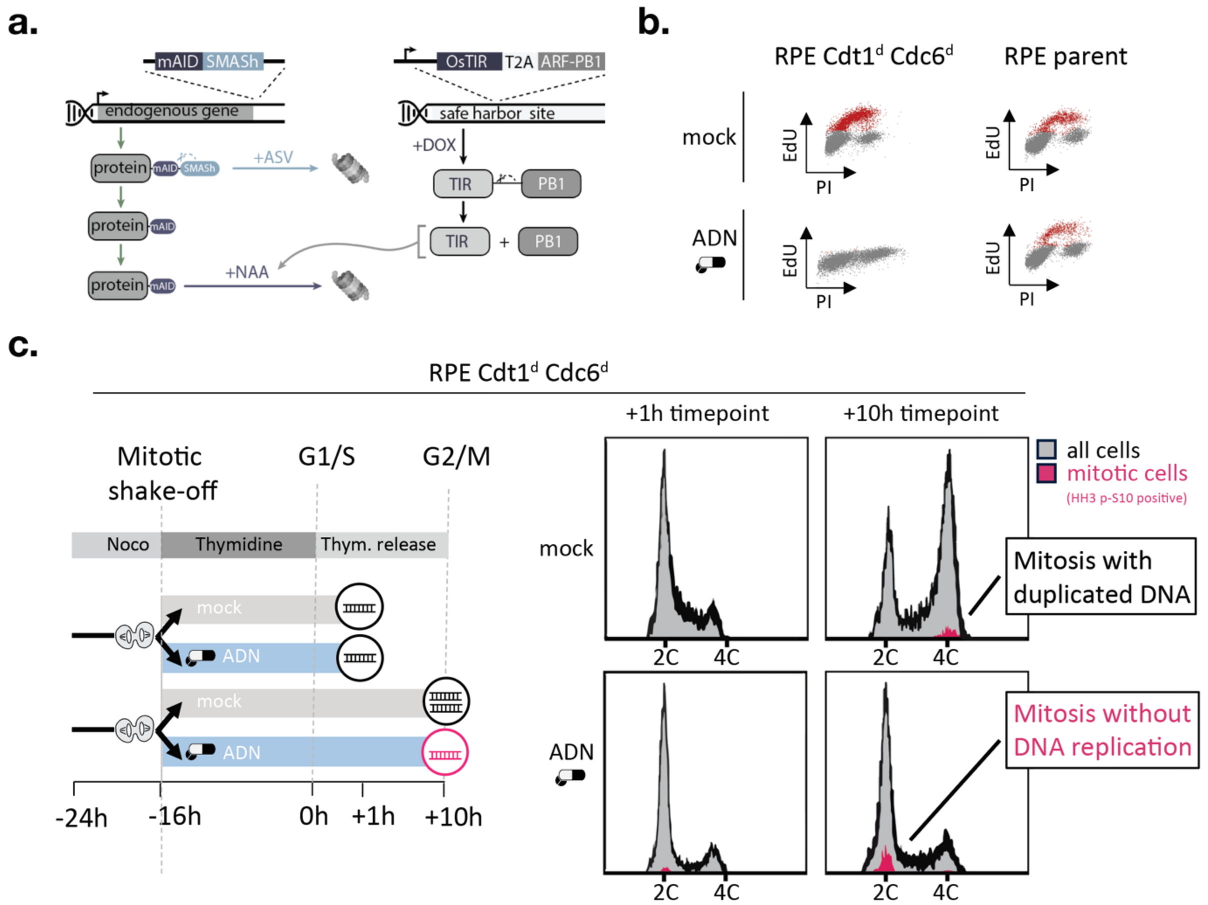

Supplementary Figure 1: Double-degron tagging of CDC6 and CDT1 allows direct and selective control of DNA replication initiation and induction of mitosis with unreplicated genomes in human RPE cells.

a, Schematic overview of double-degron system design, which is based on CRISPR-mediated tagging of endogenous genes, such as CDC6 and CDT1 with a mAID and SMASh degrons (left panel) as well as the integration of an DOX-inducible OsTIR and ARF16-PB1 co-expression construct at the human Rosa26 safe harbour locus (right panel). **b**, FACS plots of RPE Cdt1d Cdc6d cells and untagged RPE parental cells exposed to 24h ADN treatment and 1-hour EdU pulse. Cells are sorted based on DNA content by propidium iodide [1] and replication proficiency using EdU click chemistry. EdU-positive cell populations are marked in red (20.000 cells/sample). **c**, Experimental outline (left) and FACS results (right) of synchronized Cdt1d Cdc6d cells stained for DNA content [1] and a mitotic marker (HH3 S10 phosphorylation). ADN treatment results on mitotic cells with a G1-like DNA content (2C) within a single cell cycle (10.000 cells/sample).

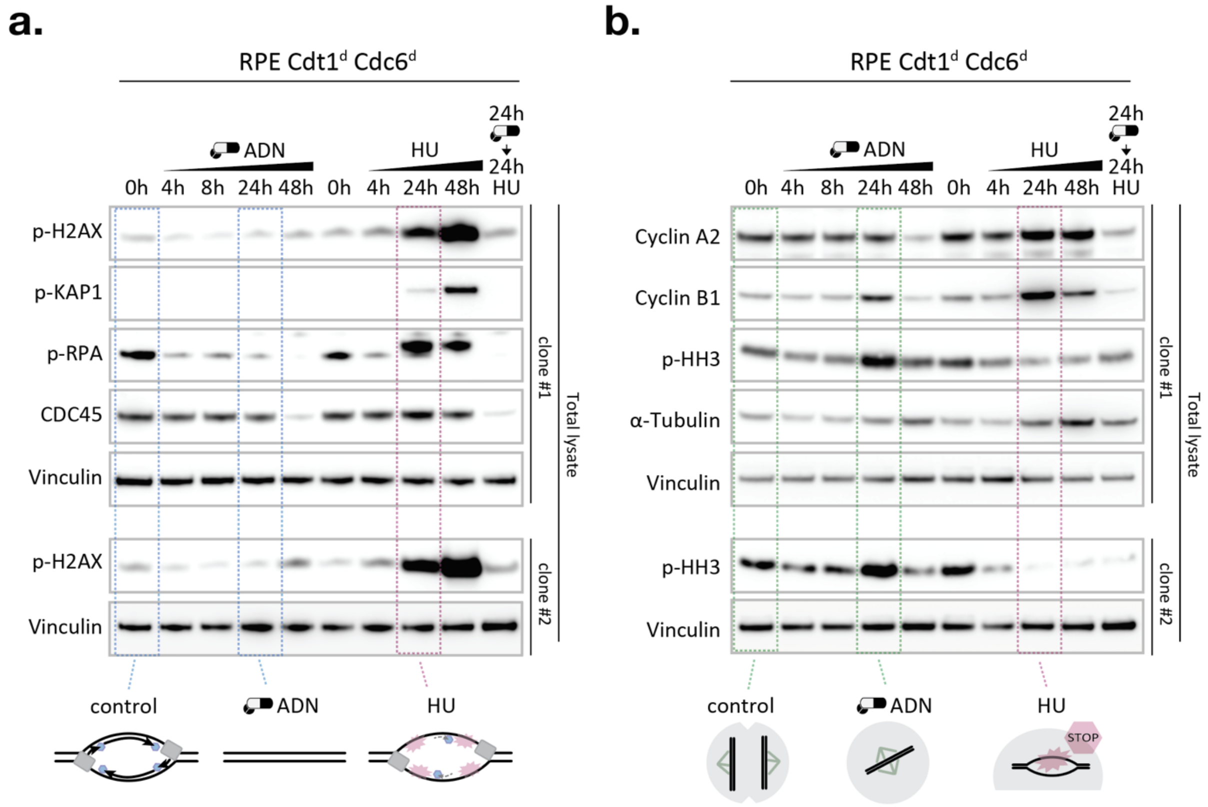

Supplementary Figure 2: CDC6 and CDT1 loss does not cause immediate DNA damage or S/G2 cell cycle arrest. a-b, immunoblots monitoring the effect of ADN treatment (indicated by drugs icon) and/or hydroxyurea (HU) using total lysate of two independent RPE Cdt1d Cdc6d lines. Histone H2AX phosphorylation served as an indicator for DNA damage (**a**), Histone H3 phosphorylation served as a marker for mitotic cells (**b**) and Vinculin served as loading control. Data are representative of two independent experiments yielding similar results. Models of the molecular consequences of the different treatments are depicted at the bottom.

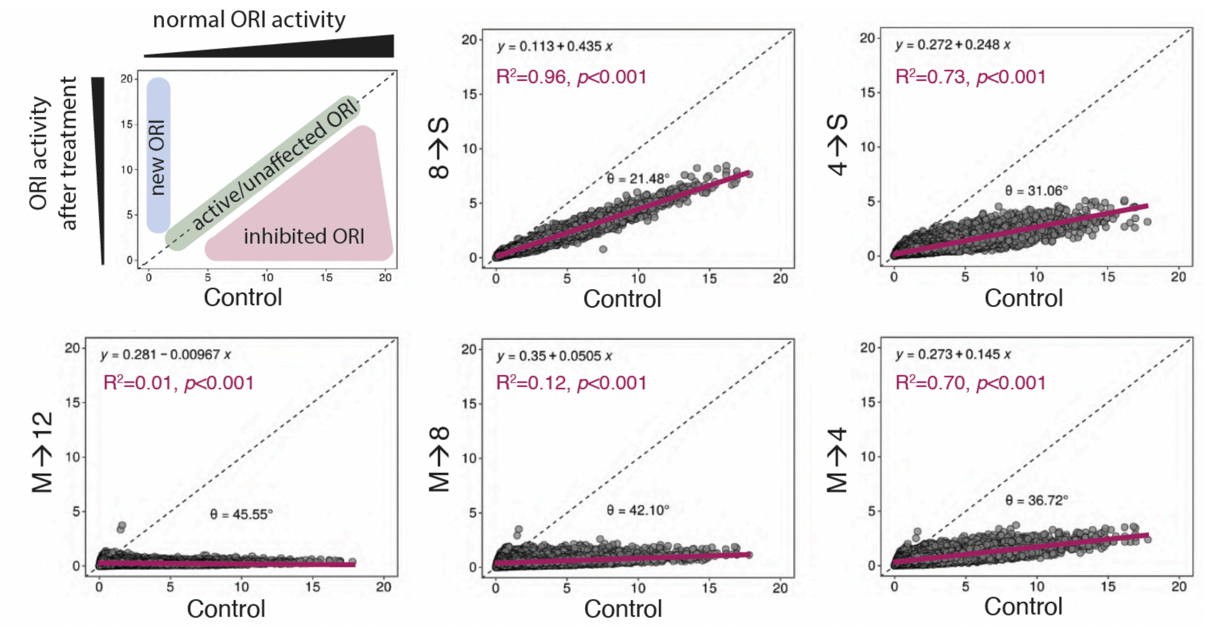

Supplementary Figure 3: CDC6 and CDT1 are critical throughout G1 to establish active origins in early S phase. Scatter plots comparing EdUseq-HU (σ) values at 1,000 individual early S-phase origins with (y-axis) or without treatment (x-axis). The treatments are performed as in Figure 2b. Upper-left graph depicts possible origin fates and their expected position on the scatter plot. To quantify the uniformity and the level of origin inhibition, correlation coefficient (R^2) between the treated and untreated data sets and the angle (θ) between the regression line and the maximum linear correlation (45 degrees) are depicted.

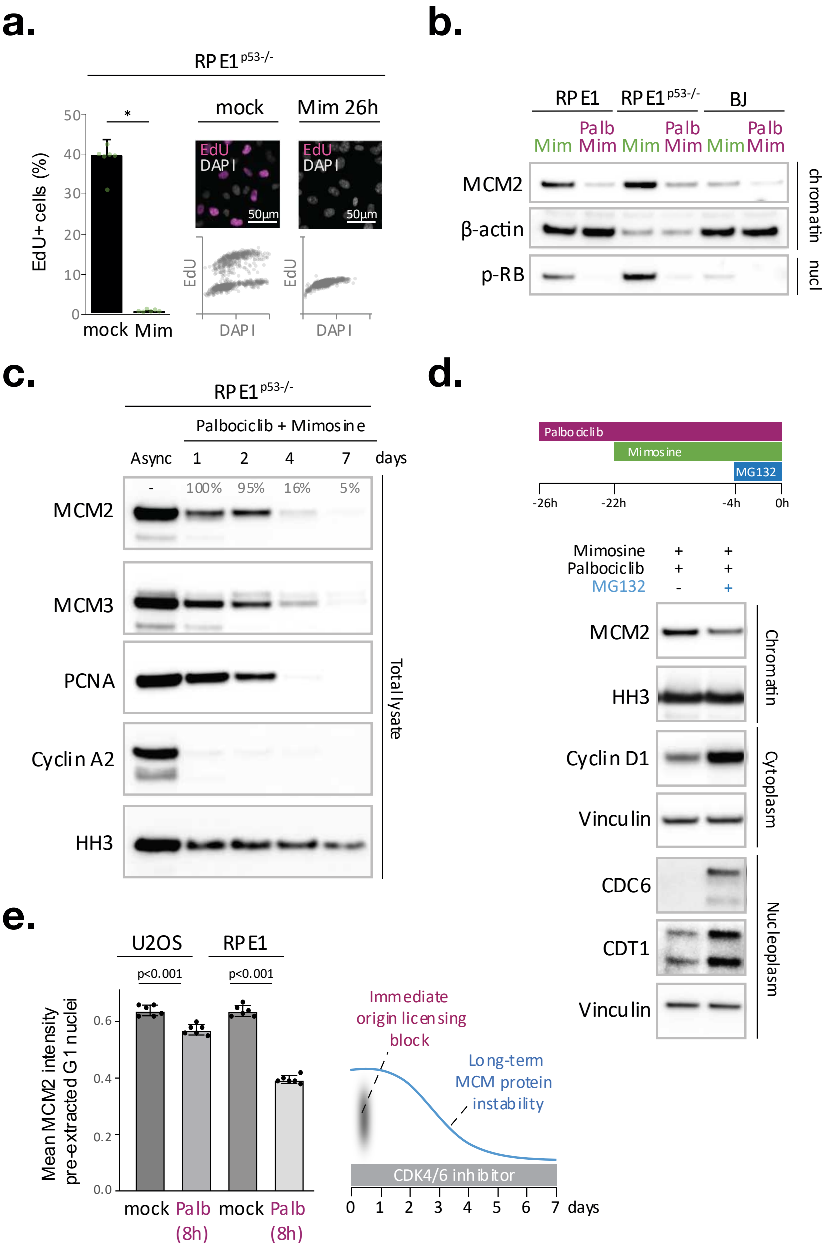

Supplementary Figure 4: Mimosine arrests cells at the G1/S phase transition and Palbociclib impedes origin licensing prior to its effect on long-term MCM protein stability. a, QIBC analysis of RPE $^{p53-/-}$ cells upon mock or Mimosine (Mim) treatment for 26 hours. Bar graph depicts average percentage of EdU-positive cells (green dots indicate replicate values, error bars indicate S.D.). Right panel depicts representative images and scatter plots of the EdU- and DAPI-stained cell populations (n=1000 cells). Asterisk indicates a significant disparity between conditions, p=0.0000000004; two-tailed paired t test; n=6 technical replicates. **b**, Experimental outline (top) and immunoblot (bottom) monitoring the effect of Palbociclib (Palb) and/or Mimosine (Min) on chromatin-bound MCM2 levels and corresponding RB (S807/811) phosphorylation levels in nucleoplasm fractions. β-actin served as internal loading control. Palbociclib addition reduced chromatin-bound MCM2 levels in three different cell lines. The experiment was repeated twice with similar results. **c**, immunoblots monitoring the long-term effects of Palbociclib and Mimosine treatment on total MCM2, MCM4 and PCNA levels in RPE $^{p53-/-}$ cells. Cyclin A2 served as a positive control for G1 synchronization by Palbociclib and Mimosine, and

Histone H3 served as loading control. Values above MCM2 bands indicate relative percentage of MCM2 signal compared to 1-day Palbociclib and Mimosine treatment, illustrating the relative stability of MCM proteins in the first 2 days of CDK4/6 inhibition. The experiment was repeated twice with similar results. The illustration below highlights the temporal order of observed events, with Palbociclib-induced origin licensing defect happening within a day and overall MCM protein instability manifesting after 2-4 days of Palbociclib treatments. **d**, Experimental outline (top) and immunoblot (bottom) monitoring the effect of proteosome inhibitor MG132 on chromatin-bound MCM2 levels. Effective proteosome inhibition was confirmed by Cyclin D1 stabilisation in the cytoplasm as well as CDC6 and CDT1 stabilisation in nucleoplasm fractions. Histone H3 and Vinculin served as loading controls. The experiment was repeated twice with similar results. **e**, Bar graph depicts mean MCM2 integrated intensities of G1 phase nuclei after mock or 8-hour Palbociclib (200nM) treatment (n=5, error bars indicate S.D., black dots indicate replicate means).

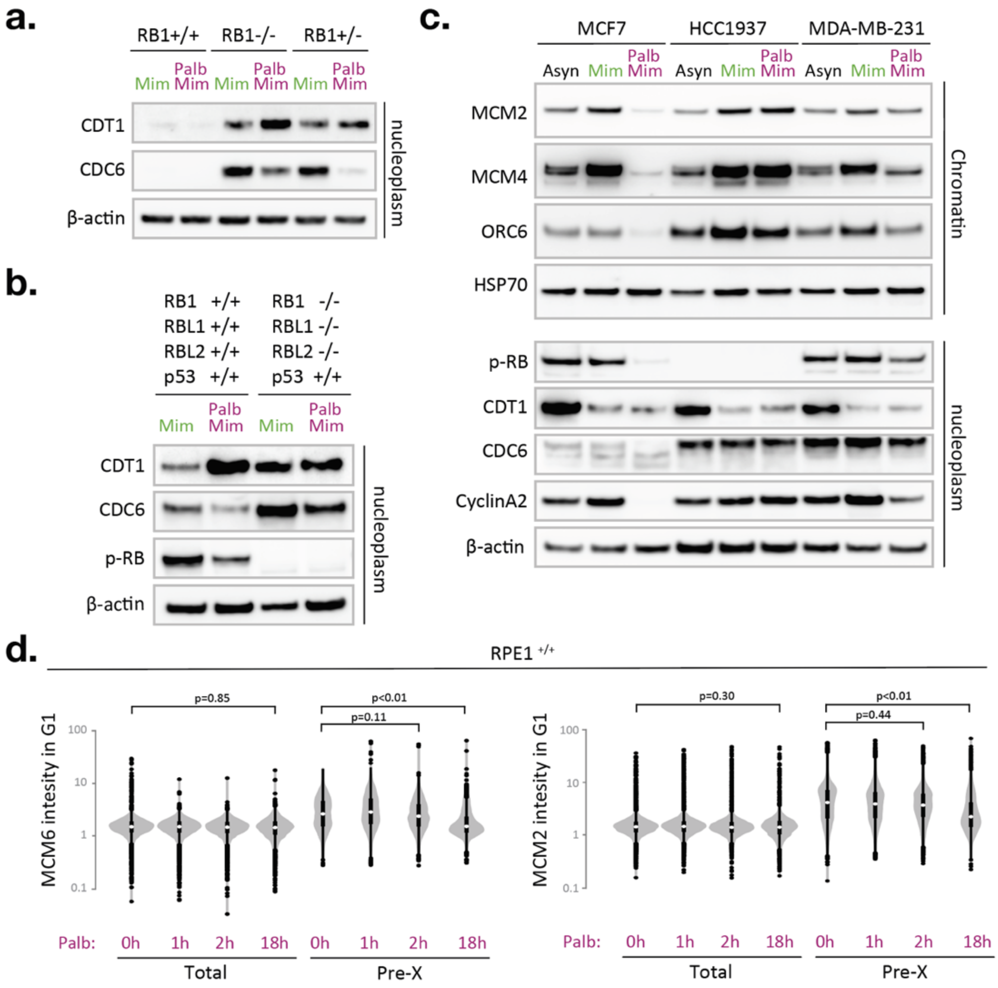

Supplementary Figure 5: CDK4/6 inhibition reduces MCM loading in RB-dependent manner but irrespective of nuclear CDT1, MCM6 or MCM2 levels. a, immunoblot monitoring the effect of palbociclib and/or mimosine on nuclear CDC6 and CDT1 protein levels in RPE cells with different levels of RB1 deficiency. Cells were treated as in Figure 3d and Figure S4b. β-actin served as loading control. Data are representative of two independent experiments yielding similar results. **b,** immunoblot monitoring the effect of palbociclib and/or mimosine on nuclear CDC6 and CDT1 protein levels in RPE cells lacking all RB pocket proteins. Cells were treated as in Figure 3e and Figure S4b. β-actin served as loading control β-actin and phospho-RB served as an internal control for palbociclib treatment and RB loss. Data are representative of two independent experiments yielding similar results. **c,** immunoblots examining the effect of 200nM palbociclib on origin licensing and nuclear CDC6, CDT1 and phospho-RB (S807/811) levels in three different cancer cell lines. Cells were treated as in Figure 3d and Figure S4b. HSP70 and β-actin served as loading control. While chromatin-association of MCM2, MCM4 and ORC6 correlate to phospho-RB levels in RB-proficient MCF7 and MDA-MB-231 cells, origin licensing is not affected by Palbociclib in RB-deficient HCC1937 cells. **d,** Violin plots depict distribution of nuclear MCM6 signals (left graph) and nuclear MCM2 signals (right graph) signals in pre-extracted (pre-X) or non-pre-extracted (Total) G1 phase RPE1 cells. G1 phase nuclei were classified based on DAPI levels. Indicated p-values represent Welch's t-test on 4 independent replicates means. Two hours of palbociclib treatment lowered chromatin-bound MCM6 levels in a subset of G1 cells, but this effect was not statistically significant in the entire G1 population.

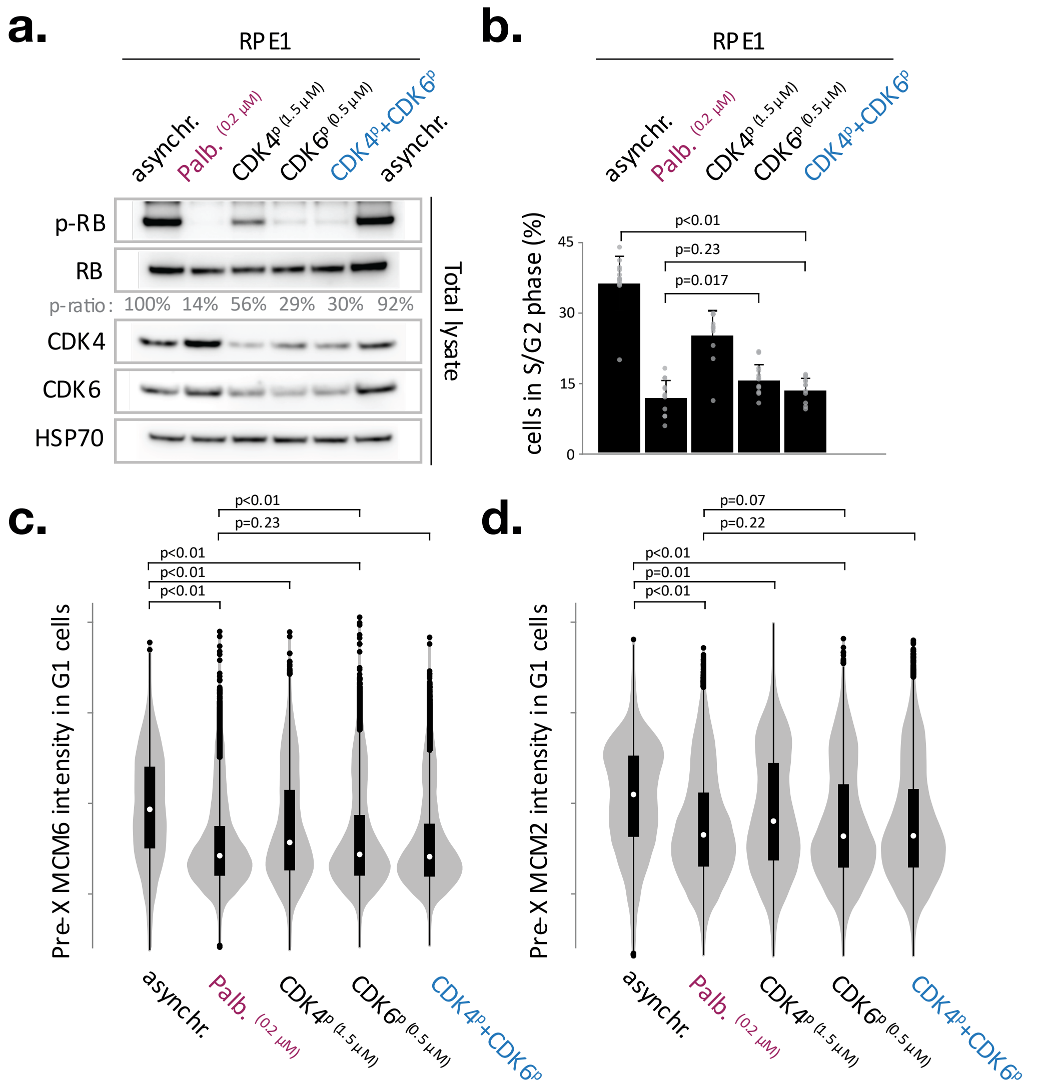

Supplementary Figure 6: Selective CDK4/6 PROTACS stall origin licensing in human RPE1 cells
a, immunoblot examining RB phosphorylation (S807/811) after 18 hours of CDK4/6 targeting using indicated concentrations of Palbociclib, BSJ-04-132 (CDK4ᵖ) and/or BSJ-03-123 (CDK6ᵖ). Data are representative of two independent experiments yielding consistent results. **b,** bar graph shows mean percentage of RPE1 cells in S/G2 phase determined by QIBC. Cells were treated as in **a**. and indicated p-values represent Welch's t-test on 12 independent replicates means. Replicate values are indicated with grey dots. **c,** Violin plots depict distribution of chromatin-bound MCM6 intensities in pre-extracted G1 phase nuclei. RPE1 cells were treated as in **a.** and G1 phase nuclei were classified based on DAPI levels. Indicated p-values represent Welch's t-test on 12 replicates means. **d,** Violin plots depict distribution of chromatin-bound MCM2 intensities in pre-extracted G1 phase nuclei. RPE1 cells were treated as in **a.** and G1 phase nuclei were classified based on DAPI levels. Indicated p-values represent Welch's t-test on 12 replicates means. We noted a high concordance between p-RB levels, percentage of S/G2 cells and MCM2/6 loading in G1 cells among the different conditions.

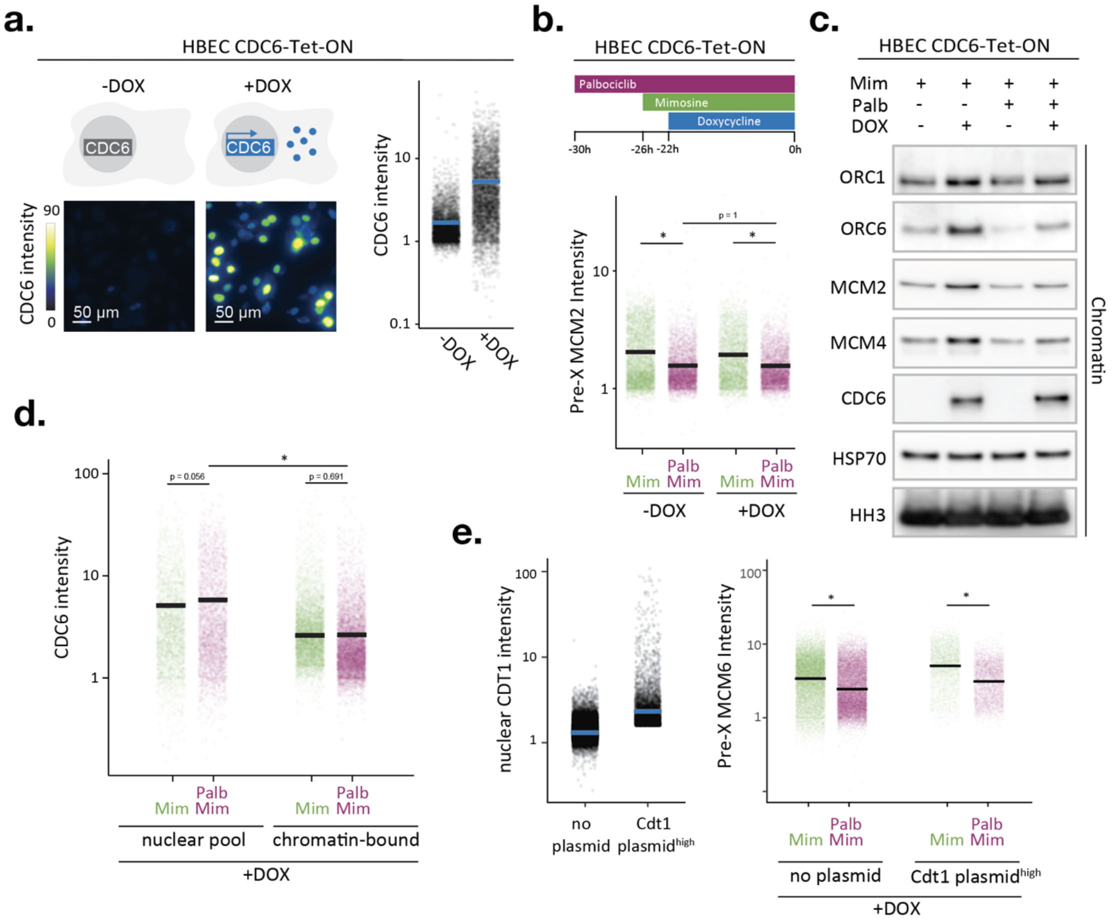

Supplementary Figure 7: Palbociclib reduces origin licensing despite CDC6 overexpression and without altering CDC6 chromatin retention in human bronchial epithelial cells. **a**, schematic overview of HBEC CDC6 Tet-ON cell model and validation of doxycycline-induced CDC6 expression. Lower panels show representative IF images confirming expected nuclear and cytoplasmic CDC6 expression patterns in single cells. Column scatter plot shows QIBC results after 22 hours of doxycycline retreatment; blue line indicates average integrated intensity of nuclear CDC6 (n=5 replicates). **b**, Upper panel shows outline of the experiment and column scatter plots indicate integrated intensity of chromatin-bound MCM2 in pre-extracted nuclei; black line indicates average integrated intensity of five replicates; asterisk indicates p<0.01 (Wilcoxon test). **c**, immunoblots monitoring protein retension in chromatin extracts after the treatments depicted in **b**; HSP70 and Histone H3 served as loading controls. Data are representative of two independent experiments yielding similar results. **d**, Column scatter plot depicts integrated intensity of nuclear or chromatin-bound CDC6 in single nuclei after the treatments depicted in **b**; black line indicates average integrated intensity of five replicates; asterisk indicates p<0.01 (Wilcoxon test). **e**, Left column scatter plot depicts integrated CDT1 intensity in single nuclei 48h post mock or Cdt1 plasmid transfection. To control for transfection efficacy and study cells in which Cdt1 expression was not limited, we selected high Cdt1 expressers for analysis (i.e. above 75th percentile of control). Right column scatter plot depicts integrated intensity of chromatin-bound MCM6 in single pre-extracted nuclei 48h post mock or Cdt1 plasmid transfection; black line indicates average integrated intensity of 3 replicates; asterisk indicates p<0.01 (Wilcoxon t-test).

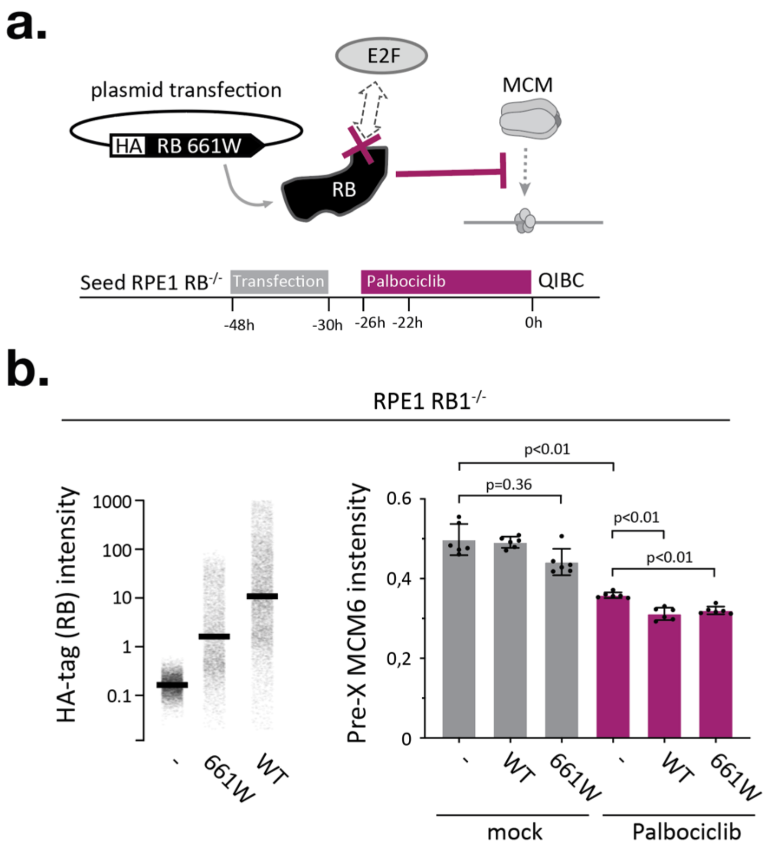

Supplementary Figure 8: Transient expression of wildtype and mutant^{661W} RB supports the reduction of MCM loading in Palbociclib-treated RB1-deficient cells. a, Schematic outline of the complementation experiment to study the role of RB-E2F in restricting MCM loading **b**, Left column scatter plot shows nuclear HA-tag signal intensities of individual cells 48 hours post transfection; black lines indicates average integrated intensities (n=6 replicates). Right bar graph shows mean integrated intensity of chromatin-bound MCM6 in pre-extracted G1 phase nuclei 48 hours post transfection; grey bars indicate mock-treated samples and purple bars indicated samples exposed to 200nM Palbociclib 26 hours prior to fixation. G1 phase nuclei were classified based on DAPI levels. Indicated p-values between conditions represent Welch's t-test on five replicates means (black dots). While the expression levels of mutant RB^{661W} were lower than RB^{wt} , the former was equally potent in inhibiting MCM6 loading in Palbociclib-treated cells, implying that RB-E2F interactions do not play a major role in immediate origin licensing suppression.

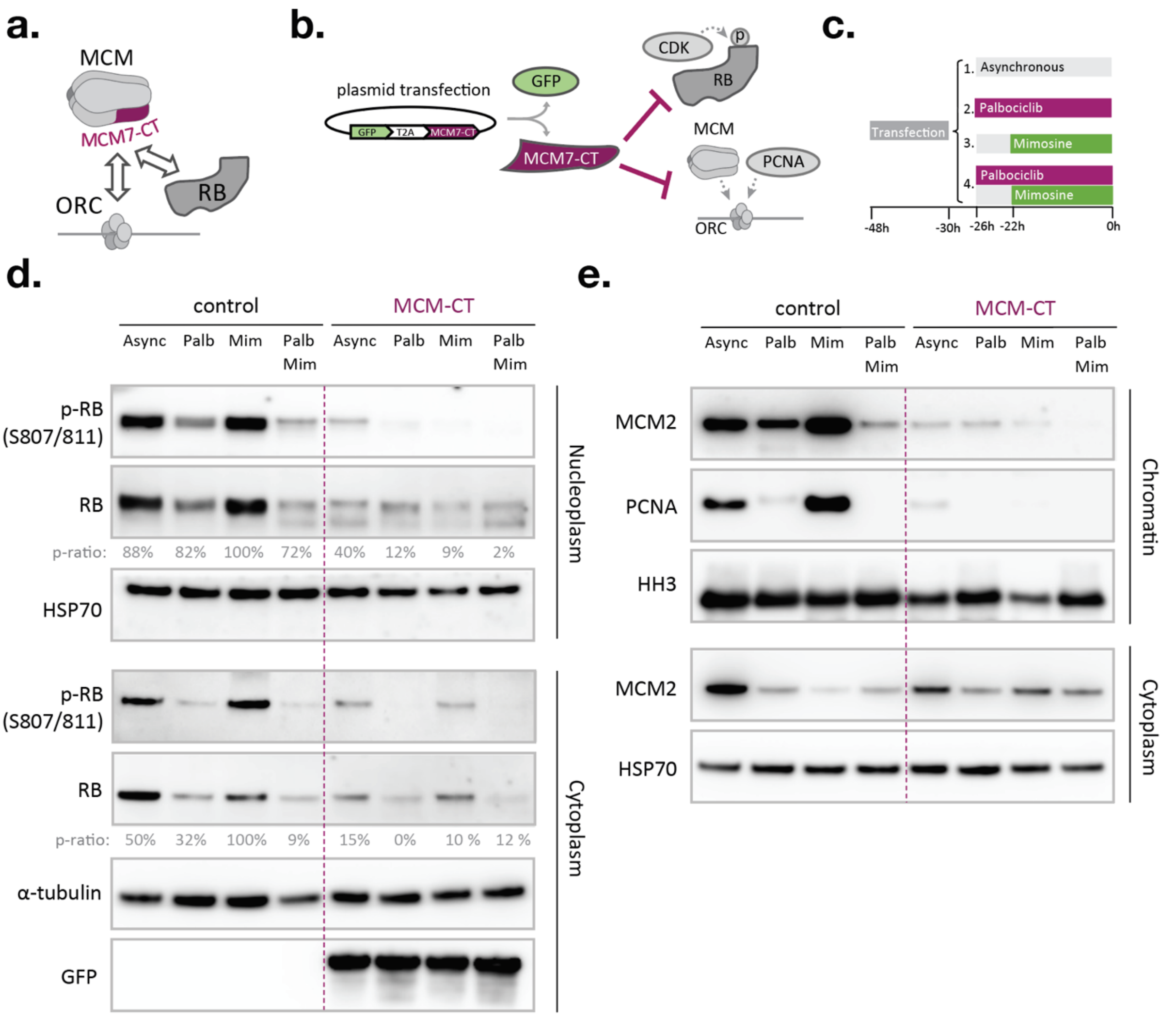

Supplementary Figure 9: Overexpression of a C-terminal MCM7 peptide prevents RB phosphorylation and origin licensing. a, Overview of reported direct interactions between ORC[2], RB[3] and the C-terminus of MCM7 (MCM-CT). **b**, Approach the study the consequences of MCM-CT overexpression; plasmid co-expressing GFP and MCM-CT was cloned and transfected in untransformed human RPE1 cells and subsequently effects on RB phosphorylation and MCM/PCNA chromatin recruitments were assessed by Western blot on cellular fractions. **c**, outline of the experiment. **d**, immunoblots monitoring the effect of MCM-CT overexpression on RB status in nucleoplasm/cytoplasm fractions. HSP70 and α-tubulin served as loading controls and GFP served as a positive transfection control. Percentages show the relative RB phosphorylation ratios, i.e., phospho-RB (S807/811) divided by total RB signal, relative to the Mimosine only control (100%). MCM-CT expression consistently reduced phosphorylation of both the nuclear and cytoplasmic RB protein pools. **e**, immunoblots monitoring the effect of MCM-CT overexpression on MCM2 in chromatin and cytoplasm fractions. Histone H3 and HSP70 served as loading controls. Data are representative of two independent experiments yielding similar results. Loss of chromatin-bound MCM2 upon MCM-CT expression was paralleled by increased cytoplasmic MCM2 levels, implying that MCM-CT caused impaired origin licensing independent of general MCM2 protein stability.

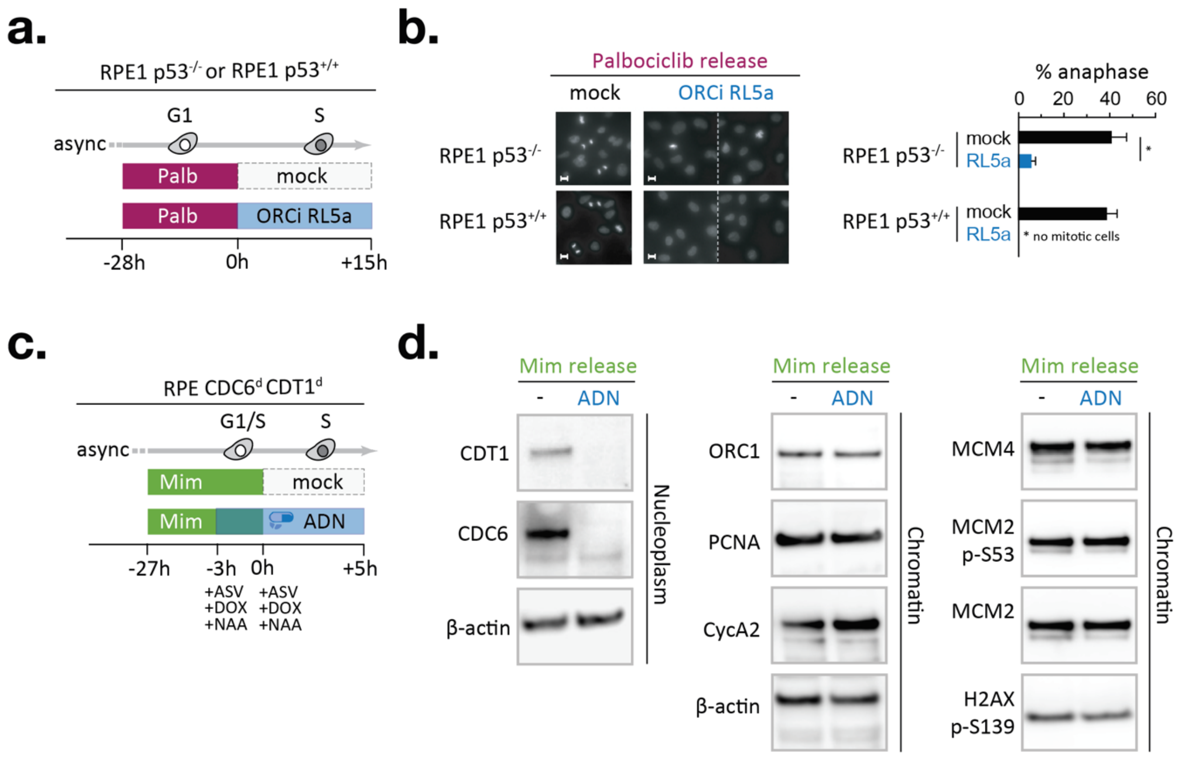

Supplementary Figure 10: The licensing defect in CDK4/6 inhibited cells can be sustained by small molecule drugs and is not due to G1/S synchronisation per se. a-c, Treatment with origin licensing inhibitor RL5a upon palbociclib release causes mitosis with unreplicated DNA specifically in p53-deficient cells; **a,** outline of experiment setup. **b,** phenotypes upon treatment described in **a**; left panel depicts representative DAPI images of p53-proficient and p53-deficient RPE1 cells; scale bars, 10 µm; right bar graph depicts quantification of the percentage of anaphase nuclei among mitotic cells; error bars indicate SD. Asterisk indicates $p<0.01$ (Student's t test; n=3). **c-d**, CDC6 and CDT1 degradation upon Mimosine release does not change chromatin occupancy of ORC1 or PCNA, nor does it impede phosphorylation of MCM complexes, which contrasts the biochemical signatures observed upon Palbociclib release (Figure 3e); **c**, outline of experiment setup. Because mimosine arrests cells in late G1 phase the release time into S-phase was shortened compared to **a**; to nevertheless ensure blocked licensing upon drug release degron drugs were added three hours before mimosine release. **d**, immunoblot of nucleoplasm and chromatin fractions upon treatment described in **c**; β-actin served as loading control. Data are representative of two independent experiments yielding similar results.

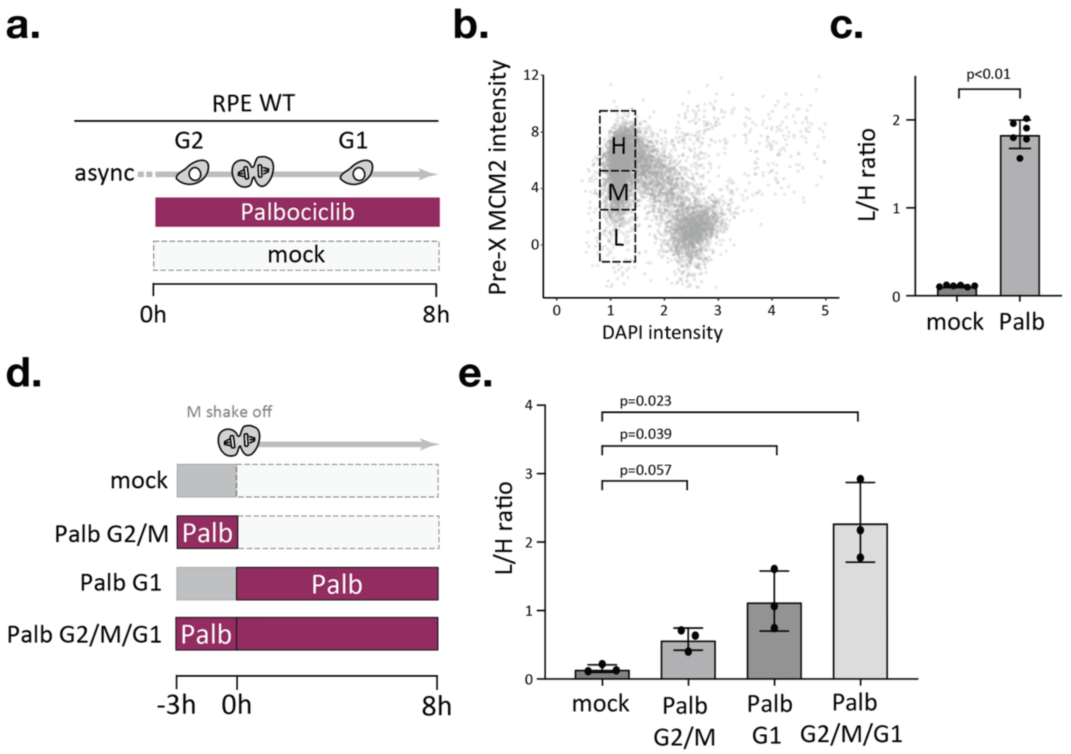

Supplementary Figure 11: CDK4/6 inhibition upon G1 phase entry impairs MCM loading. a, outline of the experiment in asynchronous RPE1 cells. **b**, Graph depicts classification of high (H), mid (M) and low (L) MCM states in G1 phase nuclei based QIBC analysis monitoring chromatin-bound MCM2 and DAPI levels in single unchallenged RPE1 cells. **c**, Bar plot shows the mean ratio between low and high MCM states (L/H ratio) in RPE1 cells treated as outlined in **a**, (n=6, error bars indicate S.D., black dots indicate replicate means). **d**, outline of the experiment in synchronized RPE1 cells. **e**, Bar plot shows the mean ratio between low and high MCM states (L/H ratio) in RPE1 cells treated as outlined in **d**, (n=3, error bars indicate S.D., black dots indicate replicate means).

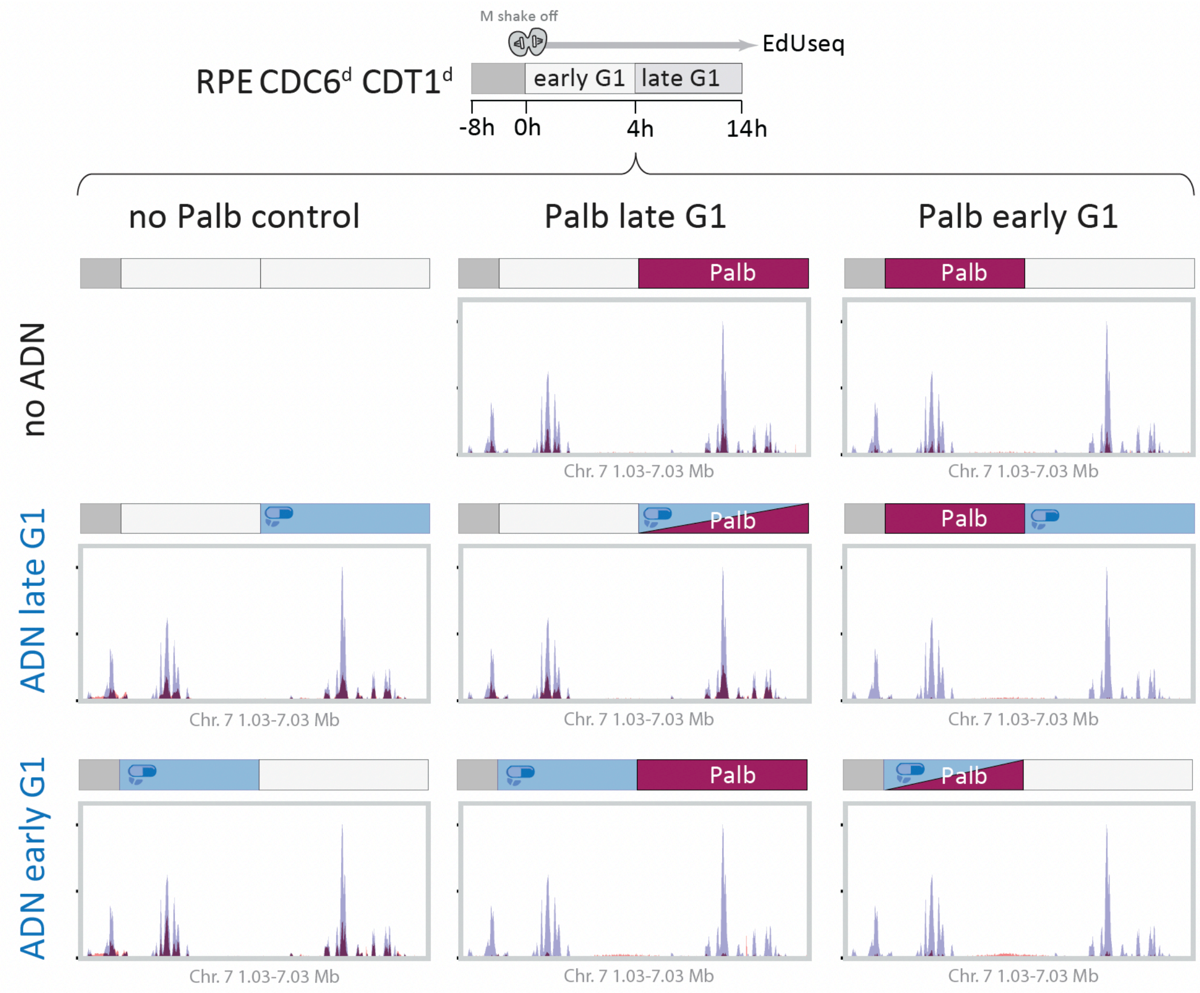

Supplementary Figure 12: EdUseq signals on a representative region of chromosome 7 in synchronized RPE CDC6d CDT1d cells treated with Palbociclib and/or ADN at different times during G1 phase. Upper panel shows general experimental setup defining two treatment windows: early G1 phase (from mitosis till 4 hours post release) and late G1 phase (from 4 hours post release till 16 hours post release).

The different treatment combinations are depicted below the accolade and for each condition replication initiation profiles (EdUseq-HU) at chromosome 7 (1.03-7.03 Mb) are shown. EdU signals post treatment (red peaks) are compared to the mock-treated control (blue peaks) and overlapping signals are shown in purple. Y-axes show EdU signal (σ), which reflects normalized number of sequence reads per bin divided by its SD; bin resolution, 10 kb; lower tick σ =100, higher tick σ=200.

Supplementary table 1

fastq_files_used:

manuscript_figure_name	SRA_upload_seq_name
Control	no_ADN_Ctrl
M->4	ADN_M_4h
M->8	ADN_M_8h
M->12	ADN_M_12h
4->S	ADN_4h_S
8->S	ADN_8h_S
no ADN control no Palb control	no_ADN_Ctrl_no_Palb_Ctrl
no ADN control Palb late G1	no_ADN_Ctrl_Palb_4h_14h
no ADN control Palb early G1	no_ADN_Ctrl_Palb_0h_4h
ADN early G1 no Palb control	ADN_M_4h_Ctrl_no_Palb_Ctrl
ADN early G1 Palb late G1	ADN_M_4h_Ctrl_Palb_4h_14h
ADN early G1 Palb early G1	ADN_M_4h_Ctrl_Palb_0h_4h
ADN late G1 no Palb control	ADN_4h_S_no_Palb_Ctrl
ADN late G1 Palb late G1	ADN_4h_S_Palb_4h_14h
ADN late G1 Palb early G1	ADN_4h_S_Palb_0h_4h

Supplementary references

1 Mesner, L. D. *et al.* Bubble-seq analysis of the human genome reveals distinct chromatin-mediated mechanisms for regulating early- and late-firing origins. *Genome Res* **23**, 1774-1788 (2013). https://doi.org:10.1101/gr.155218.113
2 Yuan, Z. *et al.* Structural mechanism of helicase loading onto replication origin DNA by ORC-Cdc6. *Proc Natl Acad Sci U S A* **117**, 17747-17756 (2020). https://doi.org:10.1073/pnas.2006231117
3 Sterner, J. M., Dew-Knight, S., Musahl, C., Kornbluth, S. & Horowitz, J. M. Negative regulation of DNA replication by the retinoblastoma protein is mediated by its association with MCM7. *Mol Cell Biol* **18**, 2748-2757 (1998). https://doi.org:10.1128/MCB.18.5.2748

Supplementary table 2:

Antibodies used in this study

Antigen	Supplier	Catalog #	Clone	Host	Dilution
Primary Antibodies (IF)					
Pericentrin	Abcam	ab4448	Polyclonal	Rabbit	1:400
Cyclin A2	Proteintech	66391-1-Ig	4E6	Mouse	1:400
MCM2	CST	4007	D7G11	Rabbit	1:400
MCM6	Santa Cruz	sc-393618	H-8	Mouse	1:400
CDC6	Santa Cruz	sc-9964	180.2	Mouse	1:400
Primary Antibodies (WB)					
MCM4	Abcam	ab4459	Polyclonal	Rabbit	1:2000
MCM3	Santa Cruz	sc-390480	E-8	Mouse	1:400
MCM2 p-S53	Abcam	ab109133	EPR5396	Rabbit	1:400
MCM2 p-S27	Abcam	ab109459	EPR19802	Rabbit	1:400
CDC7	Santa Cruz	sc-56274	DCS-341	Mouse	1:400
CDC6	Santa Cruz	sc-9964	180.2	Mouse	1:400
CDT1	Abcam	ab70829	Polyclonal	Rabbit	1:500
ORC2	Santa Cruz	sc-32734	3G6	Rabbit	1:400
ORC1	CST	4731S	7A7	Mouse	1:400
ORC6	Santa Cruz	sc-32735	3A4	Mouse	1:500
PCNA	Santa Cruz	sc-56	PC10	Mouse	1:3000
β-actin	Abcam	ab8229	AC-15	Mouse	1:10000
CDC45	Santa Cruz	sc-55569	G-12	Mouse	1:800
Cyclin A2	Novus	NBP2-67754	SD2052	Rabbit	1:1000
Cyclin D1	Abcam	ab16663	SP4	Rabbit	1:400
Phospho-RB (S807)	CST	8516	D20B12	Rabbit	1:800
RB	BD	554136	G3-245	Mouse	1:400

Histone H3	Abcam	ab1791	Polyclonal	Rabbit	1:20000
H2AX p-S139	Abcam	ab11174	Polyclonal	Rabbit	1:800
Phospho-KAP1 (S824)	Bethyl Lab	A300-767A	Polyclonal	Rabbit	1:1000
Phospho-RPA2 (S33)	Bethyl Lab	A300-246A	Polyclonal	Rabbit	1:2000
Vinculin	Abcam	ab129002	EPR8185	Rabbit	1:20000
Cyclin B1	Santa Cruz	sc-245	GNS1	Mouse	1:800
Histone H3 p-S10	Abcam	ab14955	Polyclonal	Rabbit	1:1000
α-tubulin	Abcam	ab176560	EPR13478	Rabbit	1:400
HSP70	Thermo F	MA3-007	A5A	Mouse	1:5000
Secondary Antibodies (IF)					
AF555 anti-Mouse IgG	Life Techn.	A21422	Polyclonal	Goat	1:800
AF488 anti-Rabbit IgG	Life Techn.	A11008	Polyclonal	Goat	1:800
Secondary Antibodies (WB)					
HRP anti-Mouse IgG	Sigma	A6154	Polyclonal	Goat	1:2000
HRP anti-Rabbit IgG	Sigma	A9044	Polyclonal	Rabbit	1:2000
HRP anti-Goat IgG	Abcam	ab205723	Polyclonal	Donkey	1:500
