# Supplementary material for: Temporal control of human DNA replication licensing by CDK4/6-RB signalling and chemical genetics
Source: Nat Commun. 2025 Sep 12;16:8268. doi: 10.1038/s41467-025-63669-8 (PMC12432183; doi:10.1038/s41467-025-63669-8)
Supplement: Supplementary file 2 — Reporting Summary [file 41467_2025_63669_MOESM2_ESM.pdf]

nature portfolio

Corresponding author(s): Lemmens, Halazonetis, Bartek

Last updated by author(s): Aug 8, 2025

Reporting Summary

Nature Portfolio wishes to improve the reproducibility of the work that we publish. This form provides structure for consistency and transparency in reporting. For further information on Nature Portfolio policies, see our Editorial Policies and the Editorial Policy Checklist.

Statistics

For all statistical analyses, confirm that the following items are present in the figure legend, table legend, main text, or Methods section.

n/a	Confirmed	
☐	☒	The exact sample size (*n*) for each experimental group/condition, given as a discrete number and unit of measurement
☐	☒	A statement on whether measurements were taken from distinct samples or whether the same sample was measured repeatedly
☐	☒	The statistical test(s) used AND whether they are one- or two-sided<br>*Only common tests should be described solely by name; describe more complex techniques in the Methods section.*
☒	☐	A description of all covariates tested
☐	☒	A description of any assumptions or corrections, such as tests of normality and adjustment for multiple comparisons
☐	☒	A full description of the statistical parameters including central tendency (e.g. means) or other basic estimates (e.g. regression coefficient) AND variation (e.g. standard deviation) or associated estimates of uncertainty (e.g. confidence intervals)
☐	☒	For null hypothesis testing, the test statistic (e.g. *F*, *t*, *r*) with confidence intervals, effect sizes, degrees of freedom and *P* value noted<br>*Give P values as exact values whenever suitable.*
☒	☐	For Bayesian analysis, information on the choice of priors and Markov chain Monte Carlo settings
☒	☐	For hierarchical and complex designs, identification of the appropriate level for tests and full reporting of outcomes
☒	☐	Estimates of effect sizes (e.g. Cohen's *d*, Pearson's *r*), indicating how they were calculated

Our web collection on statistics for biologists contains articles on many of the points above.

Software and code

Policy information about availability of computer code

Data collection	Genomic DNA was sonicated using a Bioruptor sonicator (Diagenode). Library preparation was performed using the TruSeq ChIP Sample Prep Kit (Illumina, Cat. No. IP-202-1012). High-throughput 100-base-pair single-end sequencing was performed on an Illumina Hi-Seq 4000 sequencer. Microscopy images were acquired using an automated Nikon Ti2 ECLIPSE microscope or Zeiss LSM 780 confocal microscope. Westernblots signals were detected using HRP-conjugated secondary antibodies and chemiluminescence was detected using SuperSignal™ West Dura (Thermo Fisher Scientific, 34076) and an Amersham Imager 600 scanner.
Data analysis	Sequencing reads were aligned on the non-masked human genome assembly (GRCh37/hg19) using the Burrows-Wheeler Aligner software as described previously (Macheret & Halazonetis, Nature 2018; Macheret et al., Cell Research, 2020). Previously described custom Perl scripts were used to assign the aligned reads to 10 kb genomic bins. Sigma (σ) values were calculated as the normalized number of reads per bin divided by its standard deviation. The data were visualized using previously described scripts (Macheret & Halazonetis, Nature 2018).<br>Cell Profiler and custom R scripts were used for image analysis. Kaluza v2.1 was used for flow cytometry analysis.<br>GraphPad Prism v9.4.1 was used for statistical analysis and graphing. Figures were assembled with Adobe Illustrator CS6.

For manuscripts utilizing custom algorithms or software that are central to the research but not yet described in published literature, software must be made available to editors and reviewers. We strongly encourage code deposition in a community repository (e.g. GitHub). See the Nature Portfolio guidelines for submitting code & software for further information.

Data

Policy information about availability of data

All manuscripts must include a data availability statement. This statement should provide the following information, where applicable:

- Accession codes, unique identifiers, or web links for publicly available datasets
- A description of any restrictions on data availability
- For clinical datasets or third party data, please ensure that the statement adheres to our policy

The fasta sequencing data and associated information described in this study will be deposited in the Sequence Read Archive (SRA) and GEO Accession Number will be provided before publication. All information supporting the conclusions are provided with the paper.

Research involving human participants, their data, or biological material

Policy information about studies with human participants or human data. See also policy information about sex, gender (identity/presentation), and sexual orientation and race, ethnicity and racism.

Reporting on sex and gender	N/A
Reporting on race, ethnicity, or other socially relevant groupings	N/A
Population characteristics	N/A
Recruitment	N/A
Ethics oversight	N/A

Note that full information on the approval of the study protocol must also be provided in the manuscript.

Field-specific reporting

Please select the one below that is the best fit for your research. If you are not sure, read the appropriate sections before making your selection.

☒ Life sciences ☐ Behavioural & social sciences ☐ Ecological, evolutionary & environmental sciences

For a reference copy of the document with all sections, see nature.com/documents/nr-reporting-summary-flat.pdf

Life sciences study design

All studies must disclose on these points even when the disclosure is negative.

Sample size	No statistical methods were used to determine the sample size. All experiments were performed in triplicate (independent biological triplicates) with few exceptions of experiments that were performed in duplicates (this is mentioned in the figure legends). The specific number of cells analysed for each experiment is reported in the figure legends for main and Exteded Data Figures. The implication of CDK4/6-RB axis in origin licensing was confirmed by independent methods (e.g. EdUseq, biochemistry and IF) and multiple different cell lines were used to determine consistency of the results across cell types.
Data exclusions	No data were excluded.
Replication	For most of the experiments, at least three independent biological experiments were performed. For each experiment, detailed description of number of replicates, sample size and statistics is provided in figure legend. Similar parameters were evaluated by multiple methods. For example, we measured chromatin-bound MCM levels in fractionated lysates and in situ in single cells by automated microscopy; having similar results with all methods.
Randomization	Cell lines were split into different plates/wells and all control and experimental treatments were randomly assigned to the plates/wells.
Blinding	The investigator was not blinded. However, the selection and detection of the variables was in most cases automated (e.g. measuring immunofluorescence and flow cytometry signal intensity), to prevent any bias by the investigator.

Reporting for specific materials, systems and methods

We require information from authors about some types of materials, experimental systems and methods used in many studies. Here, indicate whether each material, system or method listed is relevant to your study. If you are not sure if a list item applies to your research, read the appropriate section before selecting a response.

Materials & experimental systems

n/a	Involved in the study
☐	☒ Antibodies
☐	☒ Eukaryotic cell lines
☒	☐ Palaeontology and archaeology
☒	☐ Animals and other organisms
☒	☐ Clinical data
☒	☐ Dual use research of concern
☒	☐ Plants

Methods

n/a	Involved in the study
☐	☒ ChIP-seq
☒	☐ Flow cytometry
☒	☐ MRI-based neuroimaging

Antibodies

Antibodies used

Antigen Supplier Catalog # Clone Host Dilution Specificity validation
Primary Antibodies (IF)
Pericentrin Abcam ab4448 Polyclonal Rabbit 1:400 IF; localization
Cyclin A2 Proteintech 66391-1-Ig 4E6 Mouse 1:400 IF; cell cycle+ localization
MCM2 CST 4007 D7G11 Rabbit 1:400 WB/IF: localization
MCM6 Santa Cruz sc-393618 H-8 Mouse 1:400 WB/IF: localization
CDC6 Santa Cruz sc-9964 180.2 Mouse 1:400 WB: degron+
localization
Primary Antibodies (WB)
MCM4 Abcam ab4459 Polyclonal Rabbit 1:2000 WB: localization
MCM3 Santa Cruz sc-390480 E-8 Mouse 1:400 WB: localization
MCM2 p-S53 Abcam ab109133 EPR5396 Rabbit 1:400 WB/IF: localization
MCM2 p-S27 Abcam ab109459 EPR19802 Rabbit 1:400 WB: localization
CDC7 Santa Cruz sc-56274 DCS-341 Mouse 1:400 WB: size
CDC6 Santa Cruz sc-9964 180.2 Mouse 1:400 WB: degron+
localization
CDT1 Abcam ab70829 Polyclonal Rabbit 1:500 WB: degron+
localization
ORC2 Santa Cruz sc-32734 3G6 Rabbit 1:400 WB: size
ORC1 CST 4731S 7A7 Mouse 1:400 WB: size
ORC6 Santa Cruz sc-32735 3A4 Mouse 1:500 WB:
localization
PCNA Santa Cruz sc-56 PC10 Mouse 1:3000 WB/IF:
localization
β-actin Abcam ab8229 AC-15 Mouse 1:10000 WB:
localization
CDC45 Santa Cruz sc-55569 G-12 Mouse 1:800 WB: size
Cyclin A2 Novus NBP2-67754 SD2052 Rabbit 1:1000 WB:
cell cycle
Cyclin D1 Abcam ab16663 SP4 Rabbit 1:400 WB:
cell cycle
Phospho-RB (S807) CST 8516 D20B12 Rabbit 1:800 WB: inhibitor/
cell cycle
RB BD 554136 G3-245 Mouse 1:400 WB: inhibitor/
cell cycle
Histone H3 Abcam ab1791 Polyclonal Rabbit 1:20000 WB:
localization
H2AX p-S139 Abcam ab11174 Polyclonal Rabbit 1:800 WB/IF: inhibitor/
cell cycle
Phospho-KAP1 (S824) Bethyl Lab A300-767A Polyclonal Rabbit 1:1000 inhibitor/
cell cycle
Phospho-RPA2 (S33) Bethyl Lab A300-246A Polyclonal Rabbit 1:2000 inhibitor/
cell cycle
Vinculin Abcam ab129002 EPR8185 Rabbit 1:20000 WB:
localization
Cyclin B1 Santa Cruz sc-245 GNS1 Mouse 1:800 WB:
cell cycle
Histone H3 p-S10 Abcam ab14955 Polyclonal Rabbit 1:1000 WB:
localization
α-tubulin Abcam ab176560 EPR13478 Rabbit 1:400 WB:
localization
HSP70 Thermo F MA3-007 A5A Mouse 1:5000 WB:
size
Secondary Antibodies (IF)
AF555 anti-Mouse IgG Life Techn. A21422 Polyclonal Goat 1:800 -
AF488 anti-Rabbit IgG Life Techn. A11008 Polyclonal Goat 1:800 -
Secondary Antibodies (WB)
HRP anti-Mouse IgG Sigma A6154 Polyclonal Goat 1:2000 -

HRP anti-Rabbit IgG Sigma A9044 Polyclonal Rabbit 1:2000 -
HRP anti-Goat IgG Abcam ab205723 Polyclonal Donkey 1:500 -

Validation	For antibodies used to monitor protein levels by IF, specificity was determined by secondary antibody-only controls and published spatial, cell cycle and chromatin loading patterns were confirmed in asynchronous controls. For antibodies used to monitor cell cycle progression or DNA damage, we examined cells treated with or without cytostatic or DNA damaging agents, resp. For antibodies used to monitor protein levels by western blot, we validated loss of the protein band upon targeted degradation, siRNA depletion or cell cycle arrest. For protein complexes (e.g. ORC or MCM) we tested multiple subunits. Specificities of the antibodies were also validated by the manufacturer.

Eukaryotic cell lines

Policy information about cell lines and Sex and Gender in Research

Cell line source(s)	hTERT-RPE1 retinal epithelial cells (ATCC; CRL-4000)<br>BJ skin fibroblasts (ATCC; CRL-2522)<br>HBEC CDC6 Tet-ON bronchial epithelial cells https://doi.org:10.1016/j.molcel.2021.10.017<br>hTERT-RPE1 p53 knockout https://doi.org:10.26508/lsa.202000980<br>hTERT-RPE1 RB1 knockout https://doi.org:10.1158/1541-7786.MCR-17-0084<br>hTERT-RPE1 RB1/RBL1/RBL2 triple knockout https://doi.org:10.7554/eLife.37868<br>hTERT-RPE1 CDC6d CDT1d double-degron cells (this study)
Authentication	Cell lines were authenticated based on PCR, western blot and morphology.
Mycoplasma contamination	All cell lines were tested regularly and found to be negative
Commonly misidentified lines<br>(See ICLAC register)	None

Plants

Seed stocks	N/A
Novel plant genotypes	N/A
Authentication	N/A

ChIP-seq

Data deposition

☒ Confirm that both raw and final processed data have been deposited in a public database such as GEO.

☒ Confirm that you have deposited or provided access to graph files (e.g. BED files) for the called peaks.

Data access links<br>*May remain private before publication.*	GSE278714 (security token: mtwpkcuwfzwlfkz)
Files in database submission	R_CTL_r1.fastq.gz = Control<br>R_E04.fastq.gz = M->4<br>R_E08_r1.fastq.gz = M->8<br>R_E12_r1.fastq.gz = M->12<br>R_L04.fastq.gz = 4->S<br>R_L08.fastq.gz = 8->S<br>CTL0_14.fastq.gz = no ADN control no Palb control<br>CTL0_4.fastq.gz = no ADN control Palb late G1<br>CTL4_14.fastq.gz = no ADN control Palb early G1<br>A040_14.fastq.gz = ADN early G1 no Palb control<br>A040_4.fastq.gz = ADN early G1 Palb late G1<br>A044_14.fastq.gz = ADN early G1 Palb early G1<br>B040_14_rep2.fastq.gz = ADN late G1 Palb early G1<br>B040_4_rep2.fastq.gz = ADN late G1 no Palb control

B044_14_rep2.fastq.gz = ADN late G1 Palb late G1

Genome browser session
(e.g. UCSC)

https://www.ncbi.nlm.nih.gov/geo/query/acc.cgi?acc=GSE278714
https://genome.ucsc.edu/

Methodology

Replicates

Two indepenent timeseries datasets were sequenced:
i) control and ADN treated 6 samples
ii) control, ADN and palbociclib treated: 9 samples

Sequencing depth

total number of reads (single end reads, 100bp reads):

R_CTL_r1.fastq.gz : 48171191
R_E04.fastq.gz : 70186207
R_E08_r1.fastq.gz : 54779684
R_E12_r1.fastq.gz : 36535610
R_L04.fastq.gz : 45079003
R_L08.fastq.gz : 37263634
CTL0_14.fastq.gz : 57587093
CTL0_4.fastq.gz : 45383422
CTL4_14.fastq.gz : 36151352
A040_14.fastq.gz : 101981620
A040_4.fastq.gz : 74965857
A044_14.fastq.gz : 42720377
B040_14_rep2.fastq.gz : 41929075
B040_4_rep2.fastq.gz : 38015181
B044_14_rep2.fastq.gz : 45874127

Antibodies

No antibodies were used for the IP. Instead nascent DNA was labeled with EdU, which was covalently linked to biotin using click chemistry and biotin-labeled DNA was extracted using Dynabeads MyOne Streptavidin C1 beads (Thermo Fisher).

Peak calling parameters

We did not call peaks. Instead we used gene annotation data (refseq from NCBI) to align the 1000 most active ORIs in early S phase.

Data quality

We did not call peaks. Our genetic analysis (based on selective degradation of replication factors CDC6 and CDT1) confirmed that the EdU-enriched signals represent bona-fide DNA replication events.

Software

The data were visualized using custom scripts that have been submitted in the supplementary data section of Macheret and Halazonetis, Nature 2018
